# Deletion of *Core 1 β3GalT-specific molecular chaperone* (*Cosmc*) in murine intestinal epithelia leads to major alterations in glycocalyx and tumorigenesis

**DOI:** 10.1016/j.jbc.2026.111319

**Published:** 2026-02-25

**Authors:** Tongzhong Ju, Yingchun Wang, Hikaru Nishio, Matthew R. Kudelka, Xiaodong Sun, Jianmei Wang, Junwei Zeng, Lina Song, Gizem Akkas, Volkan Adsay, Charles A. Parkos, Richard D. Cummings

**Affiliations:** 1 Department of Biochemistry, Emory University School of Medicine, Atlanta, Georgia, USA; 2 Department of Pathology, Emory University School of Medicine, Atlanta, Georgia, USA; 3 Department of Pediatrics, Emory University School of Medicine, Atlanta, Georgia, USA; 4 Department of Surgery, Beth Israel Deaconess Medical Center, Harvard Medical School, Boston, Massachusetts, USA; 5 Winship Cancer Institute, Emory University School of Medicine, Atlanta, Georgia, USA

**Keywords:** *C1GalT1C1*, Cosmc, colon cancer, glycocalyx, intestinal epithelial cells (IEC), knockout mice (KO), O-glycosylation, Tn antigen, T-synthase

## Abstract

Intestinal mucins have extended O-glycans comprised primarily of the common Core 1 O-glycan (Galβ1-3GalNAcα1-Ser/Thr/Tyr) and its modifications. Expression of such glycans is under control of *Cosmc* (*C1GalT1C1*) that encodes a key ER molecular chaperone required for formation of active T-synthase, a Golgi enzyme that modifies the Tn antigen (GalNAcα1-Ser/Thr/Tyr – CD175) to generate a Core 1 O-glycan. We previously observed that targeted deletion of *Cosmc* in murine intestinal epithelial cells (IEC-*Cosmc*-KO mice) resulted in dysbiosis and alteration of the microbiome. Here we report a detailed description of these mutant mice and find that IEC-*Cosmc*-KO mice, but not WT mice, express CD175 throughout the intestinal epithelia. CD175 expression is accompanied by loss of glycocalyx, shortening of microvilli, compromised MUC2, thickening of the epithelial layer, as well as generation of high levels of reactive oxygen species. The majority of IEC-*Cosmc*^*-/y*^ mice beginning at ∼3 to 9 months spontaneously developed colorectal adenocarcinomas, some with invasive features evidenced by mesenteric metastases, which were potentially associated with activation of TGFβ signaling. Thus, deletion of *Cosmc* results in expression of CD175 and loss of extended O-glycans in IEC, which is associated with dysregulation of epithelial cell surfaces, leading to spontaneous tumor development.

All animal cells generate post-translational modifications of membrane and secreted glycoproteins with N-acetylgalactosamine linked to Ser or Thr, and even Tyr residues, GalNAcα1-Ser/Thr/Tyr (Tn antigen, CD175) which is the precursor of O-GalNAc glycans, also known as mucin-type O-glycans (1, 2, 3). In vertebrates, Tn antigen (CD175) is normally converted to the ubiquitous Core 1 O-glycan (Galβ1-3GalNAcα1-S/T/Y) which arises by action of a single enzyme, the T-synthase (*C1GalT1*), whose correct folding requires its endoplasmic reticulum (ER)-localized specific molecular chaperone, Cosmc (*C1GalT1C1*) (4, 5, 6) (Fig. 1). Normal cells do not express CD175 on mature glycoproteins (7), as Core 1 is efficiently generated and extended by addition of other sugars, *e.g.,* sialic acid, N-acetylglucosamine, and fucose to generate hundreds of structurally different O-glycans. Such extended O-glycans have many biological functions, such as being key ligands for selectins involved in leukocyte trafficking (8, 9, 10, 11), CLEC2/podoplanin interactions involved in lymphangiogenesis (12, 13, 14), and as ligands for the sialic acid binding family of Siglecs (15, 16, 17). Targeted loss of *Cosmc* in mice leads to embryonic death and defects in angiogenesis (18), whereas conditional deletion of *Cosmc* in hematopoietic/endothelial cells leads to lethal perinatal hemorrhage in the majority of mice, with the few surviving mice displaying severely prolonged tail-bleeding times and macrothrombocytopenia, and dysfunctional platelets (19). We also found that targeted deletion of *Cosmc* in neurons and astrocytes in mice leads to major changes in neuronal morphology and decreased lengths of the nodes of Ranvier (20). Importantly, hypomorphic mutations in the X-linked *COSMC* gene in males lead to a congenital disorder of glycosylation (*COSMC*-CDG) and a significant developmental and cognition phenotype (21), whereas a *de novo* mutation in *Cosmc* in females leads to mosaicism and significant loss of normal O-glycans (22). CD175 and its sialylated derivative Sialyl Tn (STn) (Siaα2-6GalNAcα-S/T/Y) (CD175s) (Fig. 1) are expressed aberrantly and frequently in human carcinomas, especially pancreatic adenocarcinomas and colorectal cancers, where they can function to drive tumorigenesis, as well as affecting innate and adaptive immunity (23, 24, 25, 26, 27, 28, 29, 30, 31, 32, 33, 34, 35).

**Figure 1:**
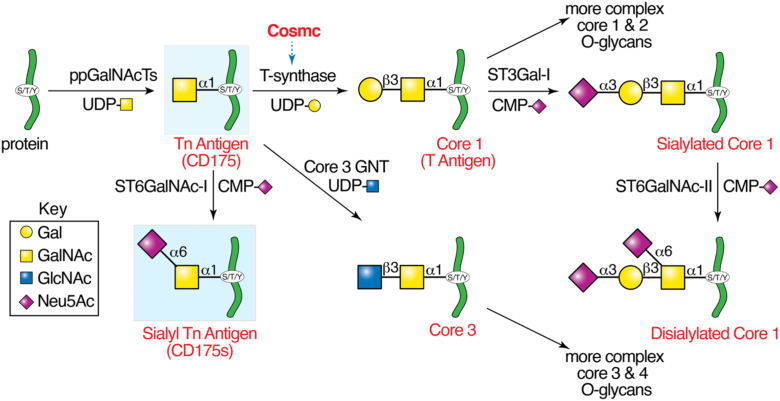
**O-glycosylation Pathway:** Mucin type O-glycosylation is initiated in the ER and Cis-Golgi by ppGalNAcTs to form the Tn antigen and completed in the Golgi after initial action of T-synthase to generate the Core 1 O-glycan. Generation of active T-synthase requires Cosmc, a unique molecular chaperone in the ER required for correct folding of T-synthase, and encoded by human Xq24 and mouse Xc3. In GI tract epithelia, Core 3 GnT (β3GnT6) can also act on Tn antigen to generate Core 3 O-glycans. Both Core 1 and Core 3 can be further extended or branched to generate Core 2 and Core 4 O-glycans, respectively. In some circumstances the Tn antigen may be modified by ST6GalNAc-I to generate the STn. STn, sialylTn antigen.

One of the major organs in which O-glycans are prominent is the intestinal tract, where epithelial cells express high amounts of mucins, including MUC2 (36, 37). In addition to Core 1 based O-glycans, O-glycans derived from Core 3 are also expressed in the intestinal tract (1, 38) (Fig. 1). We have been interested in how expression of Tn antigen in the intestinal tract might alter cellular functions and mucins. These mucins are part of the intestinal cell glycocalyx, a columnar filament network with a thickness of up to several hundred nm (39, 40, 41, 42). Such O-glycans on mucins are known to be important in regulating interactions with microbes (43, 44, 45, 46, 47, 48, 49). In this regard it is noteworthy that we observed that conditional deletion of *Cosmc* in intestinal epithelial cells (IEC) of the mouse (IEC-*Cosmc*-KO mice) leads to marked alteration of gut microbiota diversity and a proinflammatory outcome (50). Some of the same consequences have been observed in targeted deletion of T-synthase (51, 52), confirming that *T-synthase* and *Cosmc* represent genes functioning in similar pathways. However, while extended O-glycans are essential to normal development, much remains to be learned about overall functions of such glycans in development and disease.

To explore the potential consequences of *Cosmc* mutation and forced expression of CD175 on cellular functions, alterations in the cellular glycocalyx, and the role of CD175 in carcinogenesis, we explored the intestinal tract in IEC-*Cosmc*-KO (50). Here we report that *Cosmc*-KO IECs exhibit profound ultrastructural and molecular alterations in the glycocalyx and altered intestinal microvilli that contribute to development of spontaneous and invasive adenocarcinoma. These results not only reveal a fundamental role of *Cosmc* and Core 1 O-glycans in O-glycosylation to generate a normal cellular glycocalyx required for homeostasis, but also identify the Tn antigen as a contributor to tumor development.

## Results

### Molecular characterization of IEC-*Cosmc*-KO mice

**Figure 2:**
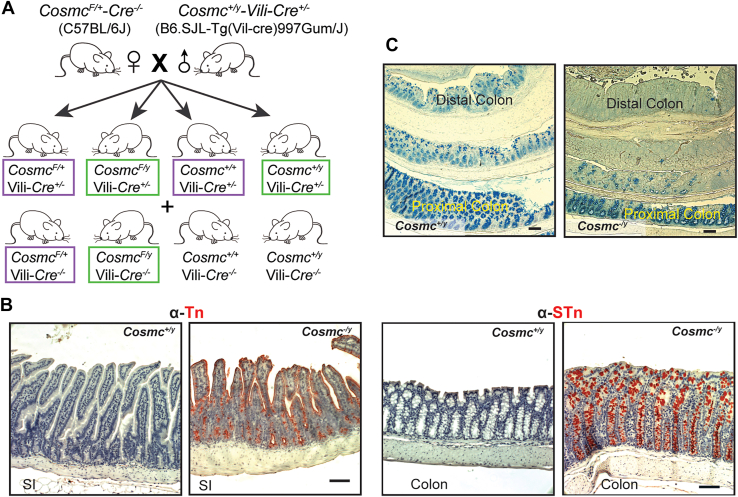
**Generation and Characterization of IEC-*Cosmc-*KO Mice:***A,* schematic illustration of the breeding strategy: *Cosmc*^*flox/+*^ female mice bred with *Cosmc*^*+/y*^-Vil-*Cre*^*+/−*^ male mice to produce *Cosmc*^*flox/y*^-Vil-*Cre*^*+/−*^ (IEC-*Cosmc-*KO, KO) mice**,***Cosmc*^*F/y*^-Vil-*Cre*^*−/−*^ and *Cosmc*^*+/y*^-Vil-*Cre*^*+/−*^ the littermate control WT mice (*green boxed*); and *Cosmc*^*flox/+*^-Vil-*Cre*^*+/−*^ (IEC-*Cosmc*^*−/+*^ mosaic)**,** and *Cosmc*^*F/+*^-Vil-*Cre*^*−/−*^ and *Cosmc*^*+/+*^-Vil-*Cre*^*+/−*^ the littermate control WT female mice (*purple boxed*). *B,* IHC of GI tract: FFPE tissue sections from GI tracts of WT and KO mice at 2∼3-M ages stained with anti-Tn and anti-STn antibodies; counterstained with hematoxylin (the bar represents 50 μm). *C, alcian blue* staining of colorectum: FFPE tissue sections from colons in a Swiss-roll from WT and KO mice at 2∼3-M ages (the bar represents 30 μm). STn, sialylTn antigen; IHC, immunohistochemistry

As reported previously (50), we generated mice with C57BL/6J congenic background harboring a *Cosmc*-deletion specifically in small and large IECs using a strategy of breeding *Cosmc*^F/+^ or *Cosmc*^F/F^ female mice (18, 19) with B6.SJL-Tg(Vil-Cre)997Gum/J transgenic male mice (Fig. 2*A*). *Cosmc*^*F/y*^:*Cre*^*+/−*^ (IEC*-Cosmc*^*-/y*^*,* IEC-*Cosmc*-KO) mice, as reported, lack Cosmc protein in purified IECs as well as being deficient in T-synthase activity (50). We immunostained for expression of the Tn and STn antigens in small intestine and large intestine of the WT and IEC-*Cosmc*-KO (IEC*-Cosmc*^*-/y*^) mice. While the Tn and STn antigens were not detected in control mice, *Cosmc*-KO IECs highly expressed Tn and STn antigens on the surface of >90% IECs and in secretion vesicles of goblet cells, but not in other cells (Fig. 2*B*). We observed a few villi and crypts in the KO mice that lacked staining for Tn/STn, likely a result from inefficient *Cre*-recombinase activity in stem cells of these villi and crypts. Cell surface and goblet cells of WT tissue sections from entire colons were stained with alcian blue (AB), a polyvalent basic dye that stains acidic glycans and mucins (53). AB staining was dramatically reduced in KO rectum, distal, and middle colon (Fig. 2*C*). Interestingly, a portion of proximal colon retained AB staining in KO mice despite expressing Tn/STn, suggesting that other types of glycans/glycoconjugates and/or core 3 type O-glycans (38, 54) may contribute to AB staining in proximal colon. Taken together, these results demonstrate specific targeted deletion of *Cosmc* in IECs leading to expression of Tn and STn antigens.

### IEC-*Cosmc*-KO mice developed rectal prolapse

**Figure 3:**
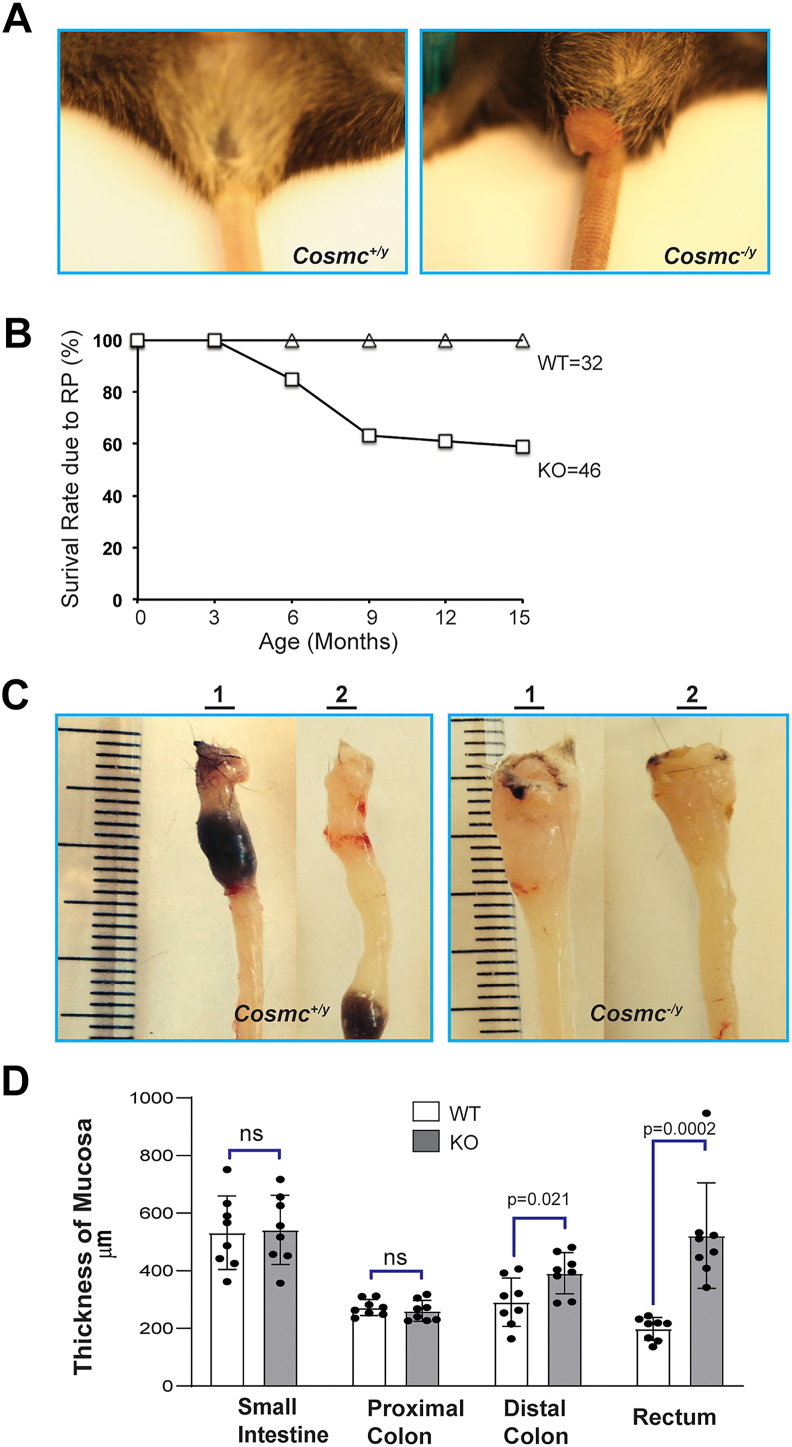
**Gross Comparison of Colons from WT and IEC-*Cosmc*-KO Mice:***A,* RP in KO Mice: KO mice suffered from RP; *B,* RP rate over time: KO mice developed RP mostly within 3∼9-M age and were terminated; the Kaplan-Meier survival curve is shown; *C,* thickened rectum in KO-mice: distal colon and rectum from two different 9-M old KO mice to that from age-matched WT mice were compared. *D,* thickness of villi and crypts (distance from tip of villus or crypt with *right* up-down orientation to the submucosa) for small intestine, proximal colon, distal colon, and rectum measured from scanned images of Swiss-roll orientated tissues with HE staining: 3 villi or crypts at similar positions from each tissue with total of 4 mice in WT and KO groups were measured and averaged (mean ± 1 SD). Statistical analysis: *t* test. RP, rectal prolapse.

Most IEC-*Cosmc*-KO mice had no gross phenotype or signs of stress and developed normally to 3 months (M) of age. There were minimal signs of colitis, including diarrhea, weight loss, or bloody stool, despite efficient deletion of *Cosmc* and robust expression of Tn/STn antigens in >90% of GI tract epithelia. A colitic score (Fig. S1, *A*–*E*) was defined by stool softness, fecal blood content, and body weight at two time points. There were significant differences, mainly in stool softness at one time point (Fig. S1*A*), but no significant difference was detected for other indices at any time point before 3M. However, between 3-9M of age, ∼40% of the KO mice developed rectal prolapse (RP), requiring euthanasia (Fig. 3, *A* and *B*). From a total of 27 KO mice at 10∼12M of age, several showed signs of distress, such as ruffled fur with moderate weight loss, and 4 mice died.

### Mutant male mice developed a spectrum of colorectal adenocarcinomas

The GI tract length was not grossly altered in IEC-*Cosmc*-KO mice and no dilation was observed in the colon; however, the rectum from KO mice was thicker than age-matched controls (Fig. 3*C*). The HE stained intestinal tissue from IEC-*Cosmc*-KO mice exhibited increased thickness of the mucosa compared to mucosa of WT mice (Fig. 3*D*). No difference was observed in small intestine and colon in young IEC*-Cosmc*-KO mice compared to WT, although the rectum exhibited consistently and significantly thickened mucosa of KO mice, which likely contributes to RP in KO mice.

**Figure 4:**
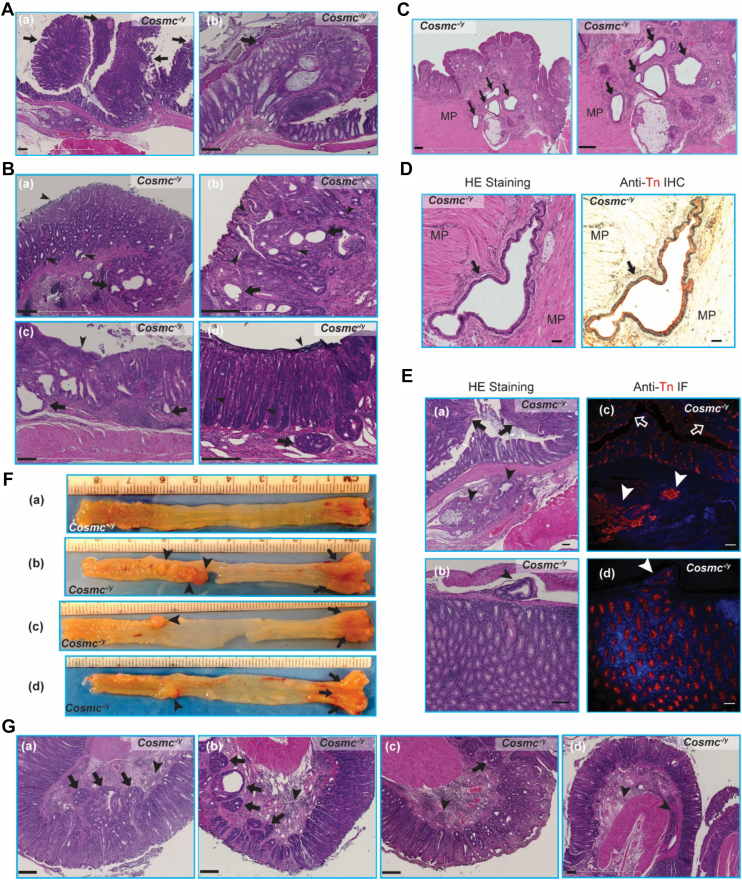
**Spectrum of Invasive CRCs Displayed in Colorectum in IEC-*Cosmc-*KO Mice:** Tissue sections of distal colon and rectum from KO mice stained with HE; representative images shown. *A,* tumors growing into the lumen seen in distal colon of a KO mouse, and surrounding epithelial cells showed dysplasia (a and b, *arrows*). The bar represents 250 μm. *B*–*D,* full spectrum of tumors in rectums from KO mice with 3∼9-M ages: cystic, atypical (B, a–d, *arrowheads*), dilated glands (B, a–d, adenoma, adenocarcinomas, *arrows*) with loss of normal crypt structure. In some cases, adenocarcinomas invaded through the SM becoming the invasive adenocarcinomas (B, a and d, *arrows*); some reached the MP, especially in ano-rectal junctions (C, *low* and *higher* magnifications). Adenocarcinoma cells appeared in MP (D, *left panel* HE staining), also expressed Tn antigen (Tn-*red*, DAPI-*blue*: nuclei) shown in adjacent tissue section (D, *rightpanel* anti-Tn IHC). The bars represents 250 μm (*B* and *C*), 50 μm (*D*). *E,* mesentery metastatic adenocarcinomas: (a and b) adenocarcinomas (HE staining) observed in lumen (*arrows*) and mesentery (*arrowheads*) from KO mice (12∼15M) (Note: The E-(a) image was derived from the same source image as A-a, but was focused on the area of metastasized tumors in the mesentery); (c and d) were adjacent tissue sections to (a and b) respectively stained with anti-Tn antibody; nuclei stained with DAPI showing both tumor cells and stroma were Tn^+^ (*arrowheads*). The rar represents 50 μm. *F,* gross tumors: the dissected tissues of distal colon and rectum from WT mice (a) and KO mice (b–d) were shown, gross tumors were observed in distal colon (b–d, *arrowheads*) and rectum (b–d, *arrows*) from KO mice. *G,* invasive adenocarcinomas developed in 3∼6-M old KO mice with RP: pathology of rectums from 4 KO mice with RP (a–d) showed loss of architectures of mucosa, atypical glands, clefts of epithelial-like cells invading through SM becoming invasive adenocarcinomas (*arrows*); some invaded into MP; many inflammatory cells infiltrated (*arrowheads*), and inflammation occurred in LP. The bar represents 250 μm. RP, rectal prolapse; IHC, immunohistochemistry; MP, muscularis propria

From a cohort of 32 WT and 46 KO mice at ages ranging from 3-18M, the GI tracts *en bloc* were analyzed for histopathologic changes. Structural organization and epithelial cell populations in the mucosa appeared normal (Fig. S2*A*). In the proximal colon, we observed no significant alteration in crypt architecture (Fig. S2*B*), yet some IEC-*Cosmc*-KO mice showed signs of increased mononuclear cell infiltration in the lamina propria that was not observed in WT mice. Inflammatory infiltrates were most pronounced in the distal colon and more so in the rectum. While ulcers were uncommon in the colonic mucosa, there were scattered foci of acute cryptitis and mucosal erosions (Fig. S2*C*). A few KO mice exhibited epithelial masses growing into the lumen around age of 12M. Histologically these masses (Fig. 4*A*-*a* and *b*, arrows) resembled tumors with altered organization of atypical epithelial cells in the distal colon.

The rectum from KO mice (*Cosmc*^-/y^) without RP displayed striking structural and cellular pathology in comparison to WT controls (*Cosmc*^+/y^) (Fig. S2*D*). Beginning at age 3M, neoplastic changes were observed in the rectum, including cellular atypia, thickening of the epithelial layer, and formation of adenomas in some KO mice (Figs. 4*B*, S2*D*). Focally, in the base of crypts, increased mitoses coupled with loss of normal cellular polarity were generally seen (Fig. 4*B*). KO mice contained atypical glands, which were abnormally branched and dilated (Fig. 4*B*, arrows and arrowheads). Significantly, in many KO mice at ages >9M, epithelial glands were observed infiltrating across the muscularis mucosa into the submucosa, a sign of invasive CRC (Fig. 4*B*-d, arrow**)**. At the anorectal junction, many *Cosmc*-KO animals had glandular infiltrates with histologic features suggestive of adenocarcinoma, also involving the muscularis (Fig. 4*C*). Some adenomas infiltrated into the muscularis propria (MP) (Fig. 4, *C* and *D*), further suggestive of invasive adenocarcinoma. Importantly, invasive tumor cells stained positively with anti-Tn antibody (Fig. 4*D*, right panel), indicating that they were derived from the *Cosmc*^-/y^ IECs. Comparing the murine colonic tumors to human colorectal carcinoma, the features were similar to well-differentiated, Duke's stage III carcinoma. Moreover, in 3/19 > 10M old KO mice, carcinomas were observed in the mesentery (Fig. 4*E*-*a*, -b) (Note: the **4E-a** panel was from the same source image as Fig. 4*A*-a but was focused on the mesentery area) and stained positively for Tn, confirming that they were derived from the *Cosmc*^-/y^ IECs (Fig. 4*E*-c, -d). The metastasized tumors in the mesentery (Fig. 4*E*-a&c, arrowheads) were likely derived from or related to the tumor masses growing into the lumen in Figure 4*A*-*a* (arrows) and Figure 4*E*-a&-c (arrows). We also observed that adjacent stroma was Tn+ in these mice (Fig. 4*E*-c), suggesting that neoplastic glands had ruptured and released mucin into the supporting stroma, as observed in human colorectal cancers (55). No metastatic tumors in liver or lung were observed in mice up to age 16M. Interestingly, while a few tumors or polyps in the colon grew into the lumen, most tumors in the rectum were flat and invaded the mucosa and MP. Inflammatory cells often infiltrated lamina propria surrounding the tumor site. Overall, all mutant mice showed variable features of neoplasia, such as hyperplasia, dysplasia, adenoma, and carcinoma (Fig. 4, *B*–*E*), and the majority (>80%) of KO mice >10M developed invasive adenocarcinoma, indicating that tumors progress through a hyperplasia-dysplasia-carcinoma sequence (Table S1). In older mice (>16M), the WT colorectum appeared normal (Fig. 4*F*-a); however, IEC*-Cosmc*-KO mice developed multiple gross tumors in the rectum and, interestingly, single or multiple tumors extended into the lumen of the proximal colon (Fig. 4*F*-b, -c and **-**d).

The observations above excluded mice with RP. However, mice that developed RP necessitated sacrifice within 24 h. These mice exhibited increased neoplastic pathology at much younger ages beginning at ∼3M, with histologic features of carcinoma consistent with a more invasive phenotype present in the colorectum compared to KO mice without RP (Fig. 4*G*). Mucosa in the distal colon and rectum appeared thicker than in control mice, which likely contributed to RP. Taken together, these results demonstrate that some IEC-*Cosmc* KO male mice spontaneously developed colorectal carcinoma.

### Distal colon and rectum IECs from IEC-*Cosmc*-KO mice are highly proliferative

**Figure 5:**
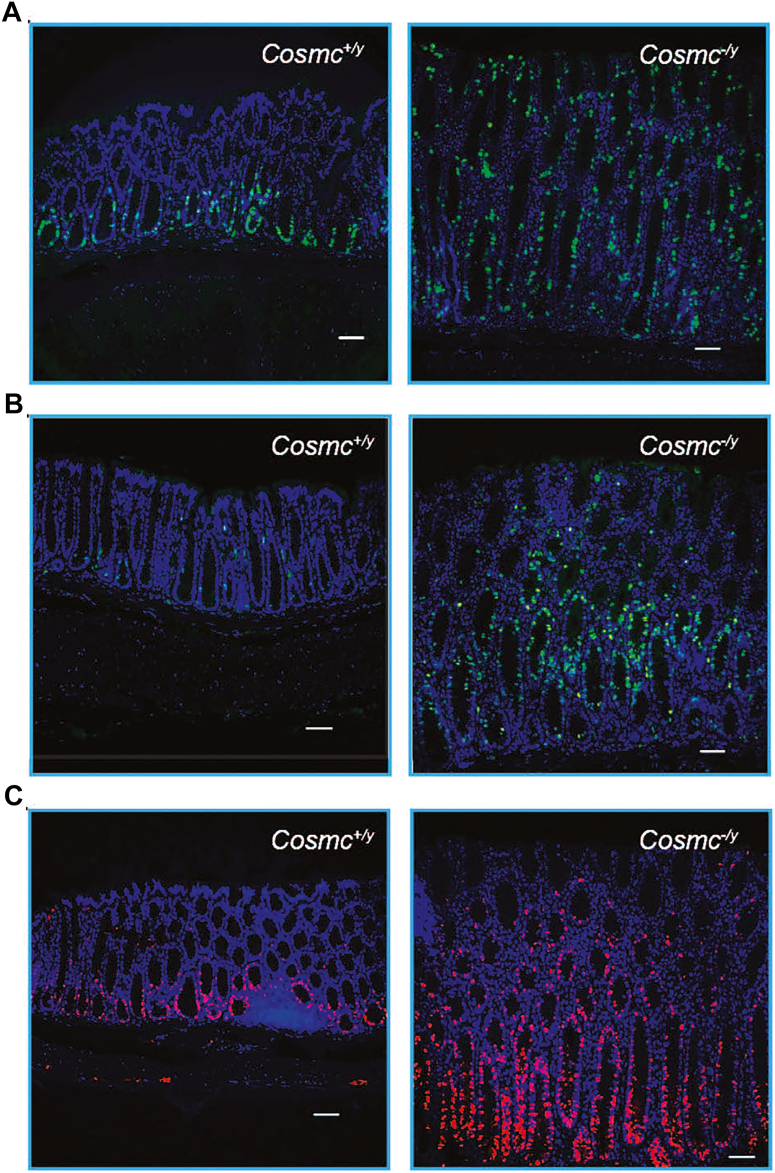
**Colorectal Tumor Cells Are Highly Proliferative:***A,* BrdU incorporation: mice at 6-M age were injected with BrdU and GI tract tissues were processed for pathology. Tissues sections were immunofluorescently stained with anti-BrdU antibody; nuclei stained with DAPI. Representative images of rectums from KO and WT mice (n = 2–3/group) are shown. *B,* Ki-67 Staining: FFPE tissue sections of GI tract from KO and WT mice were immunofluorescently stained with anti-Ki-67 antibody; DAPI used for nuclear staining. Representative images of rectums from KO and WT mice (n = 2–3/group) are shown. *C,* proliferating cell nuclear antigen (PCNA) expression: FFPE tissue sections of GI tract from KO and WT mice were immunofluorescently stained with anti-proliferating cell nuclear antigen antibody; DAPI used for nuclear staining. Representative images of rectums from KO and WT mice (n = 2–3/group) are shown. The bar represents 50 μm.

We observed that proliferation was elevated in the lower third of the crypt in control animals using BrdU incorporation. Remarkably, IEC-*Cosmc*-KO mice incorporated BrdU along the entire length of the crypt in distal colon and rectum (Fig. 5*A*), indicating enhanced proliferation, as compared to that from WT mice in which only the lower third of crypts showed incorporation of BrdU. No difference in proliferation was observed in proximal colon and small intestine (Fig. S3, *A* and *B*). Increased BrdU incorporation along the length of the crypt could result from proliferation of all cells along the crypt or enhanced proliferation of the stem cells at the crypt bottom and enhanced migration of terminally differentiated cells. Staining of Ki-67 (Fig. 5*B*) and proliferating cell nuclear antigen (PCNA) (Fig. 5*C*) were largely restricted to the lower third of the crypts in WT mice, whereas the KO mice were positive for Ki-67 and PCNA along the entire length of the crypts, indicating that most IECs in the distal colon and rectum were proliferating (Fig. 5, *B* and *C*). These data demonstrate that loss of *Cosmc* in the colorectum results in enhanced proliferation of IECs along the entire length of the crypts, associated with cellular transformation and tumor progression.

### Deletion of *Cosmc* in IECs results in loss of glycocalyx and altered microvilli

**Figure 6:**
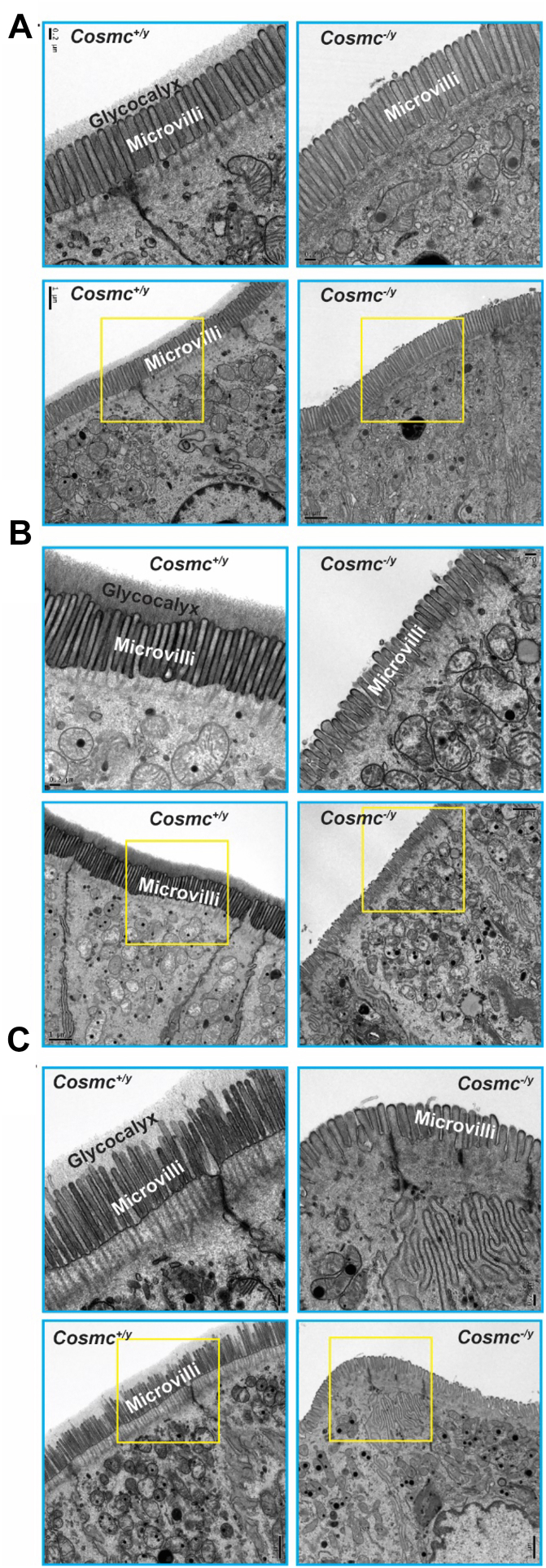
**Profound Alterations in Ultrastructure of Intestinal Epithelia from IEC-*Cosmc*-KO Mice:** GI tissues from 6-week-old mice perfused and fixed with glutaraldehyde and processed for transmission electron microscopy. Representative images of apical side of IECs shown in (*A*) Small intestine, (*B*) Colon, (*C*) Rectum. *Upper-panel* images with higher magnification (The bar represents 0.2 μm) were from boxed areas in the *lower-panel* images (The bar represents 1.0 μm). The heavy dense glycocalyx and well-organized microvilli appeared in IECs of WT (*Cosmc*^*+/y*^) mice, while IECs from KO (*Cosmc*^*-/y*^) mice lost the glycocalyx and had shorter and thicker microvilli.

Under normal conditions, the glycocalyx and associated mucins regulate epithelial homeostasis (49). The glycocalyx is comprised of specialized glycoconjugates on the apical surface (lumenal side) of intestinal epithelia and includes complex O- and N-glycans, and glycosaminoglycans. Using transmission electron microscopy (TEM) we evaluated the glycocalyx and microvilli in the small intestine (SI) (Fig. 6*A*), colon (Fig. 6*B*), and rectum (Fig. 6*C*). Controls exhibited well-organized microvilli that were ∼1.0, 1.0, 0.9 μm long in the SI, colon, and rectum (Fig. 6, *A*–*C*), respectively. The glycocalyx was also clearly detected in such controls and exhibited regional variations in thickness (SI: 0.3, colon: 0.5, rectum: 0.35 μm) (Fig. 6, *A*–*C*). By contrast, the KO mice exhibited loss of the glycocalyx in the SI and colorectum and had severely blunted microvilli (colon: 0.5, rectum: 0.5 μm) in the colorectum, but not in the SI (Fig. 6, *A*–*C*). This represents, for the first time, evidence that elimination of extended O-glycans on IECs through *Cosmc* deletion results in a profound loss of the glycocalyx and alterations in microvilli. No significant ultrastructural abnormalities were observed by electron microscopy in the appearance of tight junctions, adherens junctions or desmosomes in SI, colon and rectum, which mediate cell-cell contact.

### MUC2 was decreased in IEC-*Cosmc*-KO mice

**Figure 7:**
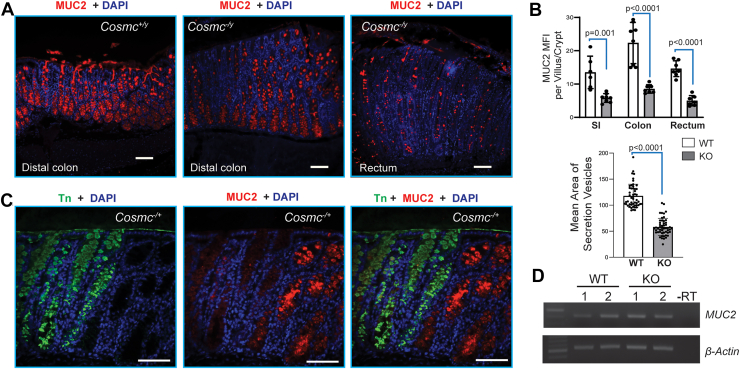
**MUC2 Decreased in KO Mice with 4∼5-M ages:***A,* immunofluorescence staining of MUC2: FFPE tissue sections of GI tracts from WT and KO mice were fluorescently stained with anti-MUC2 antibody (*red*). Representative images of distal colon from WT (*Cosmc*^*+/y*^), and distal colon and rectum from IEC-*Cosmc*-KO (*Cosmc*^*-/y*^) shown. The bar represents 200 μm. *B,* quantification of MUC2: Mean fluorescence intensities of MUC2 (*upper panel*) and the area (size) of secretory vesicles (*lower panel*) in goblet cells from colons of WT and KO mice were measured and quantified: 50-∼100 goblet cells in the same portion of GI tract from each group were chosen and fluorescence intensity was measured by NIH ImageJ Software and presented as Mean ± 1 SD. Statistical analysis: *t* test. *C,* MUC2 expression in GI tracts of IEC-*Cosmc*^+/−^ female mosaic mice: FFPE tissue sections of distal colon from IEC-*Cosmc*^+/−^ mouse immunofluorescently co-stained with anti-MUC2 antibody (*red*) and anti-Tn mouse mAb (*green*). *D,* RT-PCR*:* mRNA was isolated from IECs of 2 different WT and KO mice, and RT-PCR for *MUC2* and *Actin* were performed.

MUC2 is highly O-glycosylated and is a major mucin produced by epithelial cells in the GI tract; its loss is associated with intestinal inflammation and tumorigenesis (56). We observed overall higher fluorescence intensity of MUC2 in an IF staining with anti-MUC2 in IECs from WT than that from the KO mice (Fig. 7, *A* and *B*). We did not observe differences in the number of MUC2 expressing goblet cells in proximal colon in WT *versus* IEC-*Cosmc*-KO mice, yet the size of secretion vesicles within goblet cells in IEC*-Cosmc-*KO mice was notably smaller than in WT mice (Fig. 7, *A* and *B*), suggesting that possibly less MUC2 was produced in goblet cells in KO mice. Thus, differentiation of goblet cells from stem cells was not altered in IEC*-Cosmc-*KO mice. Importantly, we observed that the rectum of KO mice exhibited smaller size and reduced intensity of MUC2 secretion vesicles, along with fewer goblet cells, which is potentially an indicator of cellular transformation (Fig. 7*A*).

We co-stained for MUC2 and Tn antigen in colons from female IEC-*Cosmc*^*−/+*^ mice (Fig. 7*C*), exploiting the fact that these mice are mosaic due to random X-chromosome inactivation and therefore contain “patches” of Tn^+^ and Tn^−^ crypts as discussed later. We observed a remarkable mosaic pattern, in which Tn^−^ IECs (using *Cosmc*^*+*^ as the active allele) stained much more strongly for MUC2 than Tn^+^ IECs (using *Cosmc*^*-*^ as the active allele), demonstrating compromised MUC2 level in *Cosmc-null* cells (Fig. 7*C*). However, we observed no drastic difference in *MUC2* transcripts between KO and control mice as assessed by RT-PCR (Fig. 7*D*). The mechanism(s) underlying decrease in MUC2 in the KO mice is currently unknown. One of the possible mechanisms is the instability of Tn-carrying MUC2 from the KO mice, as we recently observed that the stool of IEC-*Cosmc* KO mice contains detectable Tn + glycoproteins (57).

### Elevation of TGFβ signaling in IEC-*Cosmc-*KO mice

To explore the molecular mechanisms underlying cellular transformation and tumor formation in IEC-*Cosmc*-KO mice, we investigated pathways commonly implicated in human colorectal cancer, specifically Wnt/β-catenin and TGFβ pathways. β-catenin nuclear translocation is used to assess APC/Wnt/β-catenin pathway. Although the intensity of β-catenin in epithelial cells from KO mice was higher than in WT mice, β-catenin was mainly localized to the plasma membrane and did not show significantly increased nuclear staining (Fig. S4), suggesting that this pathway was not significantly activated following *Cosmc* deletion in IECs.

**Figure 8:**
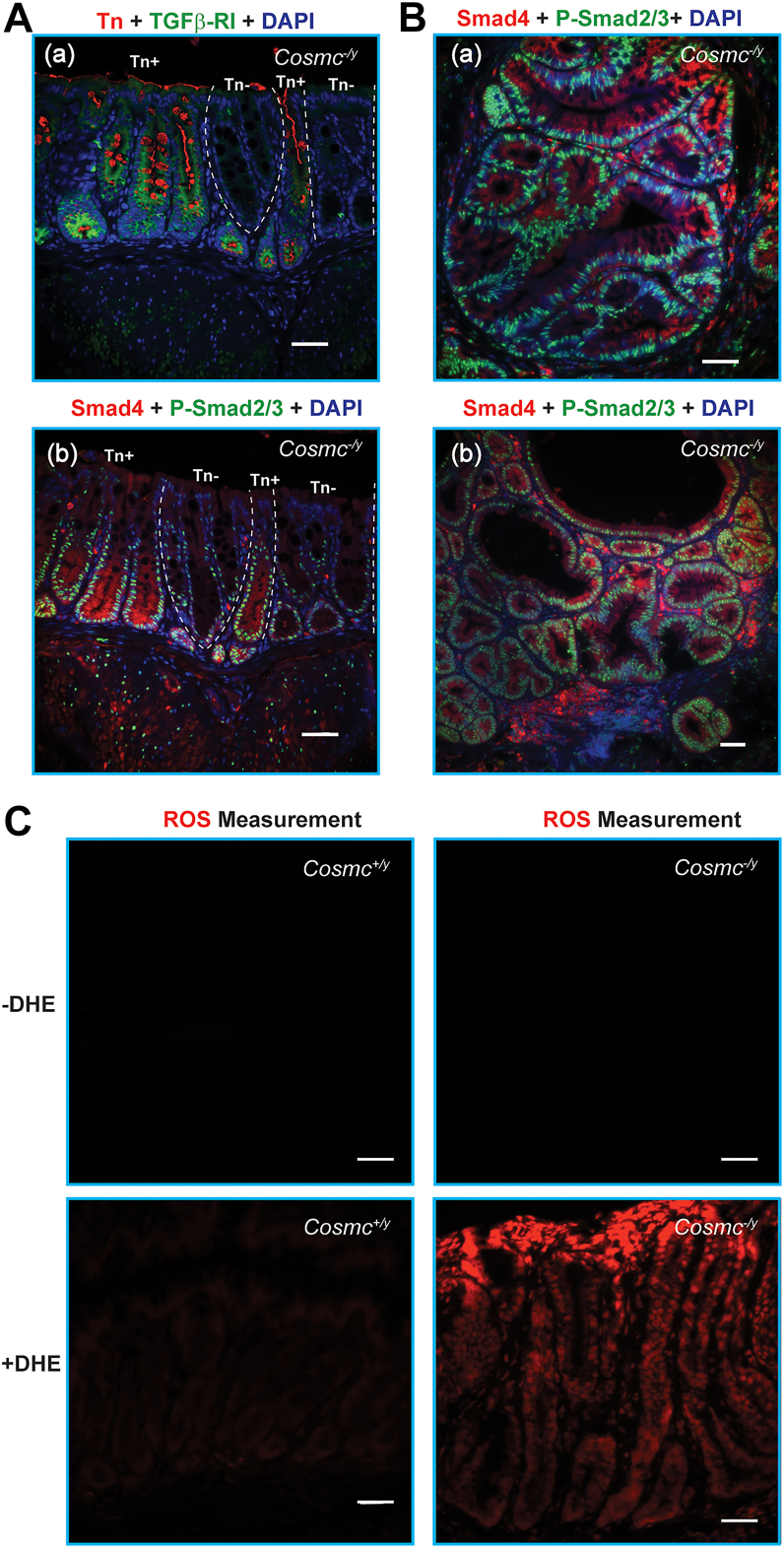
**Mechanisms for Colorectal Carcinogenesis in IEC-*Cosmc-*KO Mice:***A,* elevation of TGFβ Pathway: FFPE tissue sections of rectum from IEC-*Cosmc*-KO mice with 6∼9-M ages stained with antibodies against Tn (*red*), TGFβ-RI (*green*) (a), and an adjacent section stained with P-Smad2/3 (*green*), Smad4 (*red*) (b); nuclei stained with DAPI. The Tn^-^ crypts were bracketed in *white dashed lines*. The Tn^+^ and Tn^-^ crypts in these adjacent tissue sections are *matched* between (a) and (b). *B,* constitutive activation of TGFβ Pathway in tumor cells: tissue sections of rectum tumors were stained with P-Smad2/3 (*green*), and Smad4 (*red*); nuclei stained with DAPI. *C,* ROS increased in the rectum from IEC-*Cosmc*-KO mice: ROS (*red*) in frozen tissue sections of rectum from WT and IEC-*Cosmc*-KO mice was measured directly by incubation with dihydroethidium solution, *red* fluorescence was monitored, and images were taken post incubation. The bar represents 50 μm.

To evaluate the TGFβ pathway, we stained tissue sections for TGFβ receptors (TGFβ-RI as a surrogate) and downstream signaling molecules, phosphorylated Smad2 and Smad3 (P-Smad2/3). We examined KO mice with only hyperplasia in the rectum and distal colon, along with KO mice with invasive adenocarcinomas, in order to understand the context of altered signaling. As an internal control we identified sections in male KO mice in which a few crypts were Tn^−^ (Fig. 8*A*-a & -b), presumably resulting from inefficient *Cre*-mediated *Cosmc*-deletion. Thus, with the same experimental setting, we exploited this novel pathology to compare the activation status of the pathway in Tn^−^ *versus* Tn^+^ IECs. Furthermore, this experimental setting is ideal to investigate the Tn/STn role(s) in the normal or tumor biology because the Tn^-^ and Tn^+^ cells are living side-by-side in the same biological environment. Of note, the two rectum tissue sections for Figure 8*A*-a & -b were two adjacent sections from the same tissue block of an IEC-*Cosmc*-KO mouse, and Tn^-^ and Tn^+^ crypts in Figure 8*A*-a & -b can be matched, although not identical. Interestingly, the expression pattern of TGFβ-RI was remarkably different between Tn^−^ and Tn^+^ cells (Fig. 8*A*-a): TGFβ-RI was highly expressed throughout the entire crypts containing Tn^+^ IECs, but expressed at much lower levels in crypts containing Tn^−^ IECs (Fig. 8*A*-a). Consistently, Tn^+^ IECs exhibited enhanced P-Smad2/3 nuclear translocation and increased intensity throughout the crypts in comparison to Tn^−^ cells, with a few of them having P-Smad2/3 nuclear translocation (Fig. 8*A*-b). These results suggest that possibly constitutive activation of the TGFβ pathway occurred in Tn^+^ IECs, which could contribute to epithelial-mesenchymal transition (25, 58, 59, 60), a critical step in tumorigenesis. Furthermore, the TGFβ pathway in tumor cells was highly active as evident by intensive nuclear translocation of P-Smad2/3 (Fig. 8*B*-a, -b), suggesting that this pathway is important for tumor progression in the KO mice. As a control, Smad4 was also elevated in expression, but generally remained in the cytoplasm of Tn^+^ cells (Fig. 8, *A*-b, *B*-a, -b).

Chronic inflammation results in cytokine and chemokine production and eventually high levels of reactive oxygen species (ROS) and NOS, which cause oxidative stress, DNA damage, features that may contribute to tumorigenesis (61, 62). ROS in frozen rectum tissue sections *ex vivo* were incubated with DHE, which becomes oxidized by ROS and binds to DNA in the nucleus to generate red fluorescence (63). IECs from WT mice had minimal background fluorescence indicating low level of ROS, while *Cosmc*-KO IECs were highly labeled (Fig. 8*C*), indicating high levels of ROS in *Cosmc*-KO cells.

Taken together, deletion of *Cosmc* results in multiple and complex mechanisms of tumorigenesis, including inflammation due to the loss of protection from MUC2 and the glycocalyx, production of ROS, constitutive activation of TGFβ pathway, and likely many other factors.

### Female mosaic mice developed spontaneous adenomas in rectum

**Figure 9:**
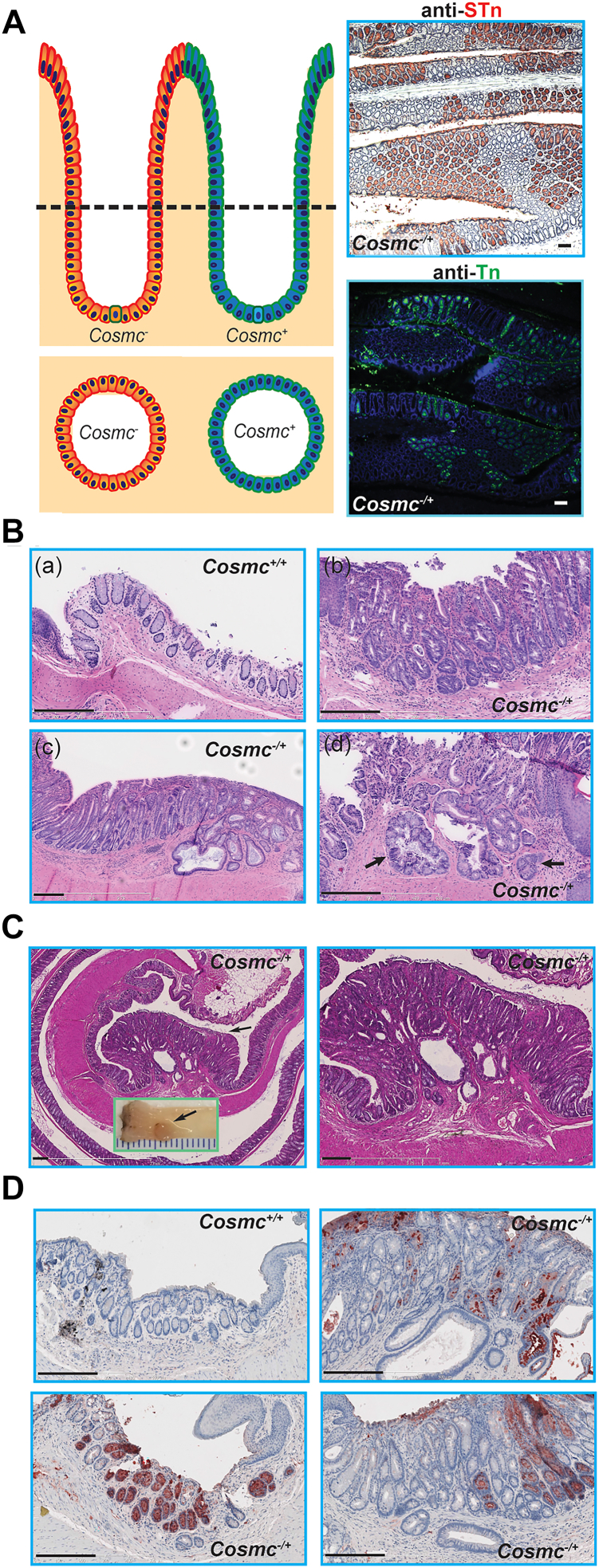
**Female Mosaic Mice Have Diverse Pathological Changes in Rectum:***A,* cartoon illustration of *Cosmc* mosaic nature of the colon IECs due to random X-chromosome inactivation (*left panel*), and tissue sections from 3-M old *Cosmc*^*+/−*^ female mice displayed the mosaicism as confirmed by anti-STn IHC and anti-Tn immunofluorescence staining (*right panel*). The bar represents 50 μm. *B,* HE stained rectal tissue: Rectum tissue sections from one WT control (a) and 3 IEC-*Cosmc*^*−/+*^ mosaic mice stained with HE, regenerative changes (b), low grade dysplasia (c) and invasive adenocarcinoma (d, *arrows*) observed in the IEC-*Cosmc*^*−/+*^ mice 11∼24-M ages. The bar represents 250 μm. *C,* rectum macro tumor in a *Cosmc*^*+/−*^ female mouse: a tumor mass growing into the lumen of rectum (inset) from a 30-M old *Cosmc*^*+/−*^ female mouse, and HE stained tissue section at lower (*left panel*) and higher (*right panel*) magnification. The bar represents 250 μm. *D,* Anti-Tn IHC: rectal tissue sections from WT control (*upper left panel*) and IEC-*Cosmc* mosaic mice (12∼15-M old) stained with anti-Tn mAb. The bar represents 250 μm. STn, sialylTn antigen; IHC, immunohistochemistry.

While male mice with *Cosmc*^*F/y*^:vil-*Cre*^*+/−*^ (IEC*-Cosmc-*KO) exhibited nearly complete deletion of *Cosmc* in all IECs resulting in Tn/STn antigen expression, heterozygous females with genotype *Cosmc*^*F/+*^:vil-*Cre*^*+/−*^ exhibit mosaicism of functional *Cosmc* due to random X-chromosome inactivation, resulting in mosaic expression of the Tn/STn antigens (Fig. 9*A*, illustration in left panel). Mosaic mice exhibit ∼50% mosaicism in IECs, about 50% of IECs with Tn and STn expression, while the other 50% IECs without Tn/STn expression (Fig. 9*A*, right panel**)**, suggesting that there is neither a significant advantage nor disadvantage for fission/expansion of Tn/STn^+^ crypts. At younger ages (<12M), the mosaic mice developed normally without significant phenotypes. Only a few of the mosaic female mice but not WT mice developed RP at >12M. Histologic analysis of mice without RP revealed changes in the colon and rectum of older female mosaic mice as observed in younger male KO mice, in particular thickening of the mucosa, and loss of normal crypt architecture, regenerative changes, atypical glands often with darker nuclear staining in comparison to WT controls (Fig. 9*B*). Furthermore, some epithelial cells or glands infiltrated across the muscularis mucosa, a sign of invasive carcinoma (Fig. 9*B*). Interestingly, a 30M old female mosaic mouse developed a tumor mass in the rectum (Fig. 9*C*, inset), which was consistent with a well-differentiated adenocarcinoma (Fig. 9*C*). In total, we evaluated 17 mosaic mice (11–30M); of these, 4 had regenerative changes, one had low-grade dysplasia, and 2 had histologic features consistent with invasive adenocarcinoma. Importantly, none of these neoplastic changes were observed in 7 WT age-matched controlled female mice. Some adenomas in mosaic female mice were Tn/STn^+^, while others were Tn/STn^−^ (Fig. 9*D*), indicating the loss of functional *Cosmc* in a portion of IECs may confer tumor transformation of Tn/STn^−^ cells, suggesting that changes in tumor microenvironments might also play a significant role in the tumorigenesis. While tumor development was significantly delayed in female mosaic mice as compared to IEC-*Cosmc*-KO males, these results demonstrate spontaneous transformation of Tn/STn^+^ epithelia in the rectum in several female mosaic mice, generally consistent with data from IEC-*Cosmc*-KO males.

## Discussion

The expression and potential role of Tn antigen in most human carcinomas, including pancreatic cancers and CRC (7, 26, 29, 30, 35, 64, 65, 66, 67, 68), has been highly enigmatic. Here we have explored a novel animal model to address this long-standing question, in which we engineered expression of the Tn antigen in IECs. The IEC-*Cosmc*-KO mice with expression of the Tn antigen in IECs exhibit major changes in epithelial glycocalyx, intestinal microvilli, hyperplasia of colonic epithelium, and constitutively develop colorectal adenoma and adenocarcinoma in addition to alterations in multiple cancer pathways. The results strongly indicate that expression of *Cosmc* is required for normal intestinal homeostasis, and that loss of *Cosmc* results in expression of the Tn antigen on glycoproteins, which is associated with altered cellular signaling and tumor initiation and progression.

IEC-*Cosmc*-KO mice spontaneously develop a full spectrum of colorectal neoplasia from hyperplasia, cellular atypia, adenoma, adenocarcinoma, invasive adenocarcinoma, recapitulating many aspects of CRC oncogenesis in regard to progression from normal epithelium to adenoma to invasive carcinoma (69, 70) (Fig. 4). Interestingly, while colon tumors tended to grow into the GI tract lumen, rectal tumors were “flat” and tended to extend into the bowel wall, consistent with activation of pathways associated with more aggressive malignant behaviors such as invasion, and possibly metastasis, despite having mostly well-differentiated phenotype. Our results are complemented by a previous study using pancreatic cancer cell lines, as well as squamous keratinocytes cultured in 2D, 3D (skin tissue equivalents), and in xenografts, which revealed multiple cancer pathways elevated or activated in *Cosmc*-mutated tumor cells expressing Tn/STn antigens on many cell surface O-glycoproteins (27).

The ultrastructural changes in IECs from IEC-*Cosmc*-KO mice are profound. Loss of *Cosmc* results in Tn antigen expression and loss of the glycocalyx (Fig. 6). This loss could arise as a direct consequence of truncated O-glycans on membrane glycoproteins/mucins of IECs or from secondary events, such as increased susceptibility to glycoprotein cleavage/digestion by microbial or host proteases in the gut lumen. Indeed, in mice in which *Cosmc* deletion was targeted to hematopoietic/endothelial cells (EHC mice), many platelet glycoproteins from such mice are defective and some are partly proteolyzed (19). Deletion of *Cosmc* was also associated with shortening of the microvilli, although the mechanism for these changes is unclear. Interestingly, we observed also that the epithelial cell junctions appeared morphologically normal.

We observed a large decrease in MUC2 glycoprotein but no drastic change in its transcripts in IECs of both IEC-*Cosmc*-KO male and *Cosmc*^*−/+*^ mosaic female mice (Fig. 7). Thus, the lack of extended O-glycans and expression of the Tn/STn antigens may be associated with the decrease of glycoprotein such as MUC2 with currently unknown mechanisms which possibly include instability and proteolysis of Tn/STn-carrying glycoproteins. Decreased MUC2 in IEC-*Cosmc*-KO mice may be a significant contributor to tumorigenesis. *MUC2*^*−/−*^ mice spontaneously develop tumors through inflammatory pathways (56), although the observed pathologies in *Cosmc*-KO mice *versus MUC2*^*−/−*^ mice is different in many ways, as discussed below.

Carcinomas in IEC-*Cosmc*-KO male mice typically developed in the distal colon and rectum, with tumors growing into the lumen in the proximal colon and multiple masses observed in SI in older mice. This regional distribution in colon corresponds to the distribution of tumors observed in CRC patients, where 60 to 70% of human CRCs are located distal to the splenic flexure, particularly in the rectum and sigmoid colon (71). Since the rectum is continuous with the colon, reasons for this anatomic preference for tumor development in humans or IEC-*Cosmc*-KO mice are not well understood. Nonetheless, IEC-*Cosmc*-KO mice exhibited several phenotypic characteristics that localized to the distal bowel in addition to other potential contributors. The mucus layer was drastically diminished in the distal colon and especially rectum, which could expose intestinal epithelia to harmful microflora that may contribute to the observed inflammation, consistent with our earlier study (50). Chronic intestinal inflammation increases the risk of CRC in both humans and mouse models. Furthermore, loss of the mucus layer likely facilitated increased mechanical damage, for example from defecation, due to reduced lubrication. This could result in a wound-repair cycle, increased proliferation, prolapse and ultimately tumorigenesis. The rectum also contains unique microflora, in part due to oxygen exposure in contrast to the colon, which may contribute to tumor localization. We reported previously that there are profound alterations in microflora in IEC-*Cosmc*-KO mice, and these changes could themselves contribute to tumorigenesis (50). Indeed, deleting *T-synthase* in IECs resulted in a shift in microbiota in the GI tract (51, 72), and also conferred colitis-associated intestinal tumor (73).

IEC-*Cosmc*-KO mice did not develop clinical signs of colitis (diarrhea, bloody stool, weight loss) in contrast to the *T-synthase* (*C1GALT1*) KO-mice, which developed colitis (52), although for the latter, colitis was reduced upon antibiotic treatment. Furthermore, an inducible *T-synthase* IEC-KO did not exhibit overt clinical signs of colitis, in contrast to constitutive KO (72). Collectively, these data suggest that environmental influences, such as microflora, may be very important modifiers of colitis in IEC*-T-synthase*-KO mice. Although no single model can fully replicate human disease, both IEC*-Cosmc* and *T-synthase*-KO mice develop intestinal inflammation, albeit with differing localization and severity, indicating that extended O-glycans are important for protecting the gut from the environment and microbiota.

It is also noteworthy that in male children with COSMC-CDG, one of the clinical features was gastrointestinal infections, which declined in frequency and severity upon reaching adolescence (21). In relation to our finding of spontaneous neoplasms in IEC-*Cosmc*-KO mice, it is interesting that *MUC2*^*−/−*^ mice develop colitis and spontaneous tumors (74, 75, 76). These arise independently from alterations of Wnt/β-catenin/Tcf4 signaling, and through an inflammation-related pathway that is distinct from and can complement mechanisms of tumorigenesis in *Apc*^*+/−*^ mice.

Collectively, intestinal homeostasis, including intestinal barrier function and integrity of the epithelial cells, are severely compromised in IEC-*Cosmc*-KO mice. Mechanistically, this is mostly due to the loss of *Cosmc* leading to an inactive T-synthase, which results in truncated O-glycans on glycoproteins and mucins. Altered O-glycosylation ultimately alters the signal transduction, glycoprotein stability/turnover in the epithelial cells, as well as impaired protections for the epithelial cells. Thus, O-glycosylation regulated by *Cosmc* plays a critical role in intestinal homeostasis.

The IEC-*Cosmc*^*−/+*^ female mice represent a potentially interesting system for studying CRC (Fig. 9). Because *Cosmc* is X-linked, random X-inactivation in mosaicism of Tn/STn expression is seen in heterozygous females. This animal line is unique in providing a self-controlled system to investigate the role of Tn/STn antigens in colorectal carcinogenesis, progression and metastasis, in conjunction with other CRC animal models, such as *APC* mutant and carcinogen-induced mice. Such mosaic expression of the Tn antigen as observed in IEC-*Cosmc*^*−/+*^ female mice may be different in each mouse of course, depending on X-inactivation status, which may contribute to the unusual phenotypes we observed.

The mechanisms for tumorigenesis in IEC-*Cosmc*-KO mice are complex. For example, Tn/STn antigens and short O-glycans on membrane O-glycoproteins including growth factor receptors, such as TGFβ-Rs and EGFR, may alter their expression or function and in turn alter signal transduction pathways (77, 78, 79). Recent studies on triple-negative breast cancer show that in Tn-positive cells there is upregulation of several O-glycosylated glycoproteins, including mmp9, ecm1 and ankyrin-2 (80).

We observed, however, that not all signaling pathways are affected, since we did not observe significant alterations of Wnt/β-catenin signaling in IEC-*Cosmc*-KO mice (Fig. S4). Aberrant O-glycosylation of membrane glycoproteins may also affect cell-cell and/or cell-ECM contacts, and elevated ROS in IECs (Fig. 8*C*) could damage many macromolecules, especially DNA (61, 62, 81). Aberrant O-glycosylation may also compromise vesicular secretion and result in subsequent cell stress because of altered secretory dynamics of O-glycosylated proteins (82). Inflammation, stress, and extracellular matrix remodeling can induce release of active TGFβ1, which may in part account for activation of the TGFβ pathway (83) (Fig. 8). These molecular disruptions might contribute to DNA damage, genomic instability, epithelial-mesenchymal transition, de-differentiation, elevated cell proliferation, cellular transformation, tumorigenesis, invasion, and metastasis. More detailed studies on molecular insights into the potential pathways, including the TGFβ pathway are needed to fully understand the mechanisms underlying the tumorigenesis in the *Cosmc*-KO mice. Nevertheless, this work supports that aberrant expression of the Tn antigen on glycoproteins/mucins may not only be involved in the progression of carcinomas but also may contribute to carcinogenesis. Association of the Tn antigen with CRC may thus represent a useful target to consider for therapeutic interventions.

## Experimental procedures

### Chemicals and reagents

Standard chemicals and BrdU (B5002) were purchased from Sigma-Aldrich. Reagents for immunohistochemistry (IHC), goat serum, and dilution buffer were from Biogenex. AEC single solution was from Invitrogen.

### Generation of experimental animals

*Cosmc*^*flox/+*^ female mice were crossed with B6.SJL-Tg(Vil-cre)997Gum/J transgenic male mice from Jackson Lab. Since *Cosmc* is X-linked, male mice carrying *Vil-Cre* will have complete deletion in the affected tissues. IEC-*Cosmc*^*-/y*^ mice were of pure genetic background of C57BL/6J. Animal studies were performed according to the approved protocol by the Institutional Animal Care and Use Committee (IACUC) at Emory University. WT, floxed-*Cosm*c, and *Vil-Cre* alleles were identified by PCR with primers listed in Table S2.

### Antibodies

All antibodies used for IHC, IF, and Western Blot are listed in Table S3. Anti-Tn monoclonal antibody, CA3638 (BaGs-6) mouse IgM, was kindly provided by the late Dr Georg F. Springer and his associate Ms Herta Tegtmeyer (Univ. of Chicago).

### Preparation of GI tract tissues

Mice were fasted overnight before euthanization with 100% CO_2_. The GI tract tissues were harvested and perfused with PBS and flushed with formalin. The tissues were fixed in formalin overnight, paraffinized for blocks in the Pathology Core Facility at Winship Cancer Institute at Emory University. Blocks were cut into 3 μm sections; at least two sections from each tissue block were used for standard HE staining.

### Measurement of mucosal thickness

Tissue sections from Swiss-Roll like orientated small intestines and colon of mice 4-6-months age were stained with HE. Slides were scanned with Nanozoomer 2.0HT and converted into digital slides. The height of up-right villi and crypts in 4 different positions in each portion of the GI tracts, small intestine, proximal colon, distal colon and rectum from 4 different mice in each group were measured, and averaged.

### Isolation of epithelial cells

Mice were euthanized and dissected, GI tract removed, flushed with TBS, and desired segment obtained. Segment was either inverted or dissected down center and cut into ∼5 cm strips. Tissue was incubated with fresh dissociation solution (30 mM EDTA, TBS, pH 7.4) at 37 °C rotating at 250 rpm for ∼30 min or until epithelium released. Solution was then pipetted over 70 μM filter (BD Falcon) and cells rinsed with TBS. Filter was inverted and epithelia freed into Petri dish with TBS and transferred to 15 ml tube, centrifuged for 10 min × 3000 rpm, and decanted. Cells were transferred with TBS to 1.7 ml Eppendorf tube, spun down as before, decanted, and snap frozen in liquid N_2_ and stored at −80 °C for later use.

### RT-PCR

The mRNA from IECs was isolated using FastTrack MAG mRNA Isolation Kit (Invitrogen, K158001), RT was performed with 5 ng of mRNA as template with a SuperScript III First-strand Synthesis System (Invitrogen, 18080-051). PCR was carried out with Phusion High-Fidelity PCR kit (New England Biolabs, E0553L) in a 25-μl reaction. For cDNA of *Cosmc*, *T-synthase*, *MUC2* and *β-Actin*, primers (Table S2) were used, and the PCR products were analyzed on a 1.5% (wt/vol) Tris-acetate EDTA agarose gel.

### Immunohistochemistry (IHC)

For immunohistochemical detection of Tn and STn antigens, deparaffinized sections were boiled for 20 min in 0.01 M citrate buffer at pH 6.0 for epitope retrieval, followed by a 20 min incubation with 0.6% H_2_O_2_ in methanol to inhibit endogenous peroxidase activity. Sections were incubated overnight at 4 °C with primary antibodies. The sections were subsequently incubated with horseradish peroxidase labeled secondary antibody for 45 min. After wash, the sections were finally incubated with AEC substrate for 30 min and counterstained with hematoxylin.

### Immunofluorescence staining (IF) and fluorescence microscopy

For immunofluorescence detection of MUC2, BrdU, Ki-67, PCNA, TGFβ-RI, Phosphorylated Smad2 and -3 (P-Smad2/3), Smad4, β-Catenin (Table S3), deparaffinized sections were boiled for 20 min in 0.01 M citrate buffer at pH 6.0 for epitope retrieval. Sections were incubated overnight at 4 °C with primary antibodies. The sections were subsequently incubated with Alexa Fluor 488 or 568–conjugated secondary antibodies (1:500; Invitrogen) for 45 min, and the nuclei were stained with DAPI (Invitrogen) for 5 min. After wash, mounting medium was added to the sections (Vector Laboratories).

### Western Blot

Cell extracts were prepared from isolated IECs as previously described and Western blot (1:500 anti-Cosmc, Santa Cruz, sc-271829; and 1:1000 anti-β-Actin, Santa Cruz, sc-8432) was performed as previously described (6).

### BrdU incorporation

Four mice at 6-months age from each WT and KO group were injected with BrdU (50 mg/kg body weight) through intra-peritoneal cavity. Post-24 h injection, mice were sacrificed, and GI tract tissues were processed as for regular pathology.

### Fecal occult blood test

Blood content in mouse feces was detected with blood test (Hemoccult SENSA, Beckman Coulter).

### TEM

GI tract tissues were fixed overnight in 2.5% (vol/vol) glutaraldehyde in 0.1 M cacodylate buffer (pH 7.2) at 4 °C. Tissues were washed with the same buffer and post-fixed in 1% buffered osmium tetroxide, dehydrated through a graded ethanol series to 100%, and embedded in Eponate 12 resin (Ted Pella). Ultrathin sections were cut on a Leica UC6rt ultramicrotome (Leica Microsystems) at 70 to 80 nm, and counter-stained with 4% (wt/vol) aqueous uranyl acetate and 2% (vol/vol) lead citrate. Sections were examined using a Hitachi H-7500 transmission electron microscope (Hitachi High Technologies of America) equipped with a Gatan BioScan CCD camera.

### ROS–DHE staining

Mice were euthanized and large intestines were immediately removed, frozen, and cryosectioned at 10 μm. Detection of superoxide (ROS) levels using dihydroethidium (DHE) was performed. Sections were incubated with 1 μmol/L DHE (Molecular Probes) in acetone in a light–protected humidified chamber at 37 °C for 30 min. After DHE loading, slides were cover-slipped, and digital images were captured by fluorescent microscopy (Olympus 1 × 51) at same exposing condition for all slides.

### Image analysis

The fluorescence images of MUC2 stained tissues were collected at the same settings. Using NIH ImageJ (https://imagej.net/ij/) Software, the mean value of integrated fluorescence density (mean fluorescence intensities) was measured for the 7∼8 intact villi/crypts at 3 different positions (SI, colon, and rectum) from WT and KO mice. A region next to crypts that had no fluorescence was also selected and set as the background. The corrected crypt fluorescence was calculated (corrected crypt fluorescence = Mean Density – Mean fluorescence of background readings). For measuring the size of secretion vesicles of goblet cells, ∼100 goblet cells of 10∼12 intact crypts at different positions were selected; the area of each MUC2 staining in those goblet cells was measured. The mean area of secretion vesicles of goblet from WT and KO mice were calculated and compared.

### Statistical analysis

Statistics were performed using GraphPad Prism Version 10.4.1 Software. Two-tailed unpaired *t* test and ANOVA with Sidak *post hoc* comparison were used for parametric analyses (colitic disease score, MUC2 mean fluorescence intensities, MUC2 area, mucosa thickness). All values are expressed as mean ± SD, except colitic disease scores, which are expressed as mean ± SEM. *p* value was individually indicated in each figure panel.

### Data availability

All data is presented in the manuscript or is available upon reasonable request.

## Supporting information

This article contains supporting information.

## Conflict of interest

The authors declare that they have no conflicts of interest with the contents of this article.
