# Supplementary material for: Deletion of Core 1 β3GalT-specific molecular chaperone (Cosmc) in murine intestinal epithelia leads to major alterations in glycocalyx and tumorigenesis
Source: J Biol Chem. 2026 Feb 25;302(4):111319. doi: 10.1016/j.jbc.2026.111319 (PMC13049941; doi:10.1016/j.jbc.2026.111319)

**Supporting Information, Ju et al.**

**Supplemental Figure Legends**

**Figure S1. Colitic Disease Score of IEC-*Cosmc*-KO Mice.** Age-matched ~2-M old IEC-Cosmc^+/y^ and IEC-Cosmc^-/y^ mice (n=5/group) were assessed for clinical indices of colitis. (**A**) Stool softness (hard = 0, soft = 2, diarrhea = 4); (**B**) fecal blood content (no blood = 0, occult blood = 2, macroscopic blood = 4); (**C**) stool index (average of stool softness and fecal blood content); (**D**) body weight were measu­­­­red on day1 (D1) and day12 (D12) in 5 mice per group; and (**E**) weight change was also assessed on D12 as compared to D1. Data expressed as mean ± SEM, with each point corresponding to an individual mouse. Statistical analyses: (**A – D**) Two-way ANOVA. P value for **A** and **C** from Sidak post hoc comparison; only significant P values from post hoc comparison shown. P value for **B** and **D** from genotype term, and (**E**) P value from unpaired, two-tailed Student’s *t*-test.

**Figure S2**. **Pathology of GI tract from IEC-*Cosmc-*KO Mice:** The GI tract tissues from IEC-*Cosmc-*KO and WT mice at 6-M were HE stained and examined pathologically. **(A)** Small intestine: IECs and villus structures from KO mice appeared normal. **(B)** Colon: IECs and crypts of proximal colon were similar to that from WT, although vesicles within goblet cells were smaller than that from WT. **(C)** Colon: Scattered acute cryptitis (arrows) resembling ulcerative colitis (UC) occurred in the colon of some KO mice. **(D)** Rectum: the mucosal membrane was much thicker in KO mice; inflammation, dysplasia, and adenoma were seen in KO animals. Bar: 50 μm.

**Figure S3. IECs in Small Intestine and Proximal Colon Were Not Significantly Different in Proliferation:** Mice at 6-M age were injected IP with BrdU (10 mg/kg). Post-24 hrs injection, mice were sacrificed and GI tract tissues were processed. Tissues sections of small intestine (**A**) and proximal colon (**B**) were immunofluorescently stained with anti-BrdU antibody; nucleus was stained with DAPI. Representative images from KO and WT mice (n=2-3/group) are shown. Bar: 50 μm.

**Figure S4. IECs Were Not Significantly Different in β-Catenin expression:** β-Catenin and DAPI staining in WT (**A**) and KO (**B-C**). The sections were immunofluorescently stained with specific antibodies as listed in **Supplementary Table 3**. The nuclei were stained with DAPI. Representative images from KO and WT mice (n=2-3/group) are shown. Bar: 50 μm.

**Supplemental Tables 1-3**

**Supplemental Table 1: Summary of Pathology in GI tract of IEC-*Cosmc*-KO Male Mice**

| **Age** | **# of Animals (RP)^#^** | **No Change** | **Dysplasia**  **(RP) ^#^** | **Adenoma**  **(RP) ^#^** | **Adenocarcin-oma (RP) ^#^** | **Metastatic Tumor** |
| --- | --- | --- | --- | --- | --- | --- |
| <3M | 3(0) | 2 | 0 | 1 | 0 | 0 |
| 4~6M | 12(7) | 1 | 1 | 1(1) | 2(6) | 0 |
| 7-9M | 12(10) | 1 | 1 | 0 | 0(10) | 0 |
| 10~12M | 7(1) (-2)* | 0 | 0(1) | 0 | 4 | (1) |
| 13~18M | 16(2) (-2)* | 0 | 1 | 0 | 12(2) | (2) |
| Total | 50(19) | 4 | 3 | 2 | 20 | (3) |

^#^: Number of mice that developed Rectal Prolapse (RP)

***:** Mice died of unknown cause, histology was not performed because of decomposition of the GI tract tissue.

**Note:** None of the 5~10 littermate control WT mice in each age group had tumors.

**Supplemental Table 2: PCR and RT-PCR primers for Genotyping**

**and Gene Expression**

| **Genes** | **Sequences of PCR or RT-PCR Primers** |
| --- | --- |
| Villin-*Cre* | 5´-GTGTGGGACAGAGAACAAACC -3´ (F)  5´-ACATCTTCAGGTTCTGCGGG-3´ (R) |
| Floxed *Cosmc* | 5´-CATCTGCCACCAGCCAGC-3´ (F)  5´-GGTCCAGCCACCAGCTTGC-3´ (R) |
| *Cosmc* | 5′-ATCACTATGCTAGGCCACATTAGGATTGGA-3′ (F)  5′-GGAGGTAAGAAAACCAATGCATCATTGAAAA-3′ (R) |
| *T-synthase* | 5′-TGAGTATTTTGTTGCGAGAAGAGGCTGC-3′ (F)  5′-AATTTCCATGCACCTTCCCAGAGCTAA G-3′ (R) |
| *β-Actin* | 5´-GTGGGCCGCTCTAGGCACCAA-3´ (F)  5´-CTCTTTGATGTCACGCACGATTTC-3´ (R) |
| *MUC2* | 5'-TGTTTCAGGCTCCATCAC-3' (F)  5'-ACGGTACAACCCATTCAC-3' (R) |

**Supplemental Table 3:** **Information on Antibodies used for**

**IHC, IF, and Western Blot**

| **Antigens** | **Species** | **Isotype** | **Dilution** | **Vendor &Catalog Number** |
| --- | --- | --- | --- | --- |
| Tn (CA3638) | Mouse | IgM | 1:200 | In House |
| STn (TAG72, B72.3) | Mouse | IgG | 1:200 | Santa Cruz;  Sc-20042 |
| Cosmc | Mouse | IgG | 1:500 | Santa Cruz; (H-10, sc-271829) |
| β-Actin | Mouse | IgG | 1:1500 | Santa Cruz;  (C4, sc-47778) |
| BrdU | Rat | IgG2a | 1:200 | Santa Cruz; (sc-70441) |
| Ki-67 | Rat | IgG | 1:100 | Dako; (TEC-3, M7249) |
| PCNA | Mouse | IgG | 1:100 | Abcam; (ab2426-1) |
| TGFβ-RI | Rabbit | IgG | 1:200 | Santa Cruz;  (sc-999) |
| Smad4 | Mouse | IgG | 1:200 | Santa Cruz;  (sc-7966) |
| MUC2 | Rabbit | IgG | 1:400 | EPITOMIC; (EPR6145) |
| β-Catenin | Mouse | IgG | 1:2000 | Sigma;  (C2206) |
| SMA | Mouse | IgG | 1:500 | Sigma;  (A2547) |
| p-Smad2/3 | Rabbit | IgG | 1:200 | Santa Cruz;  (sc-11769-R) |
|  |  |  |  |  |

**Supplemental Figures 1-4**

**Supplemental Figure 1**


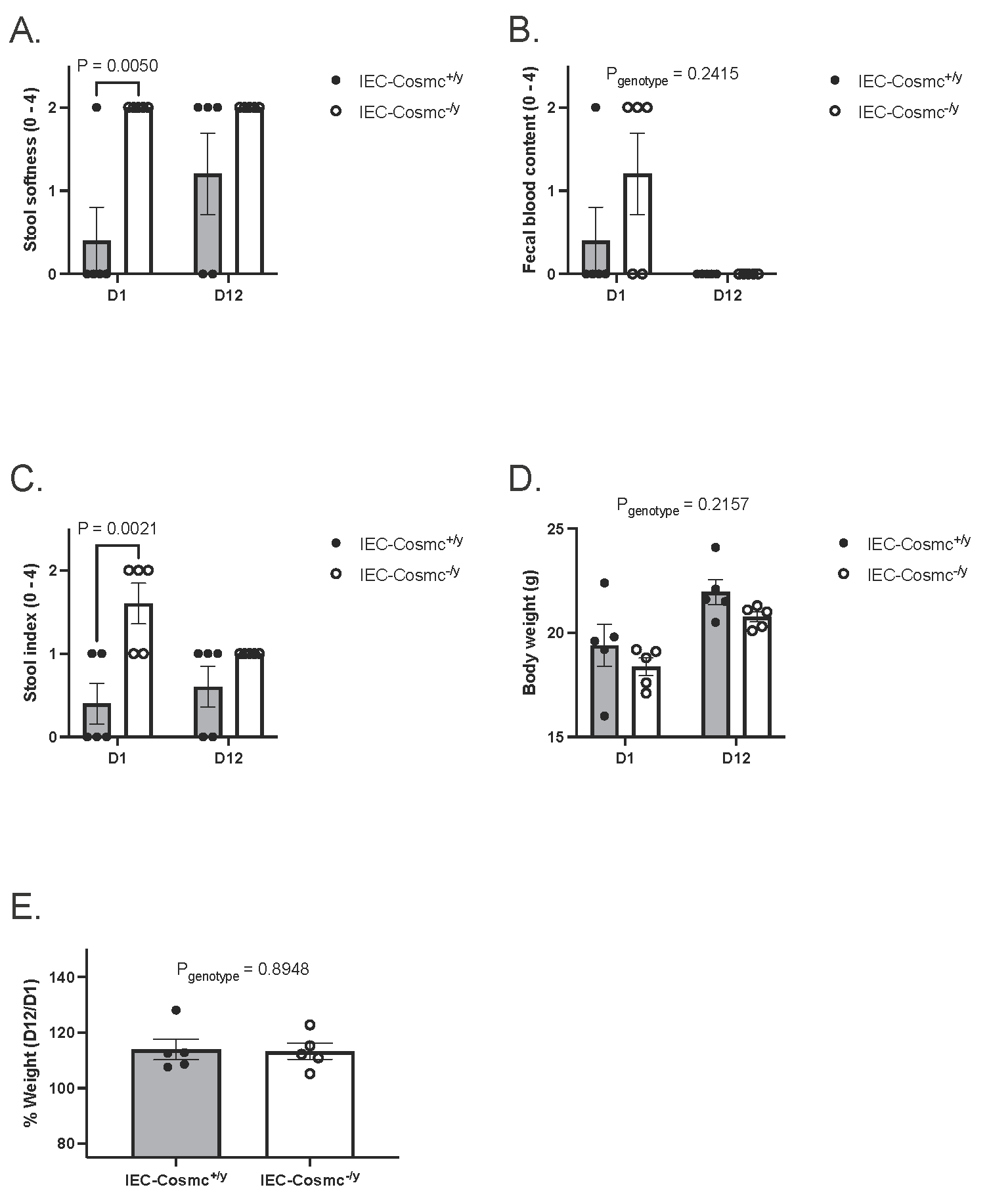


**Supplemental Figure 2**


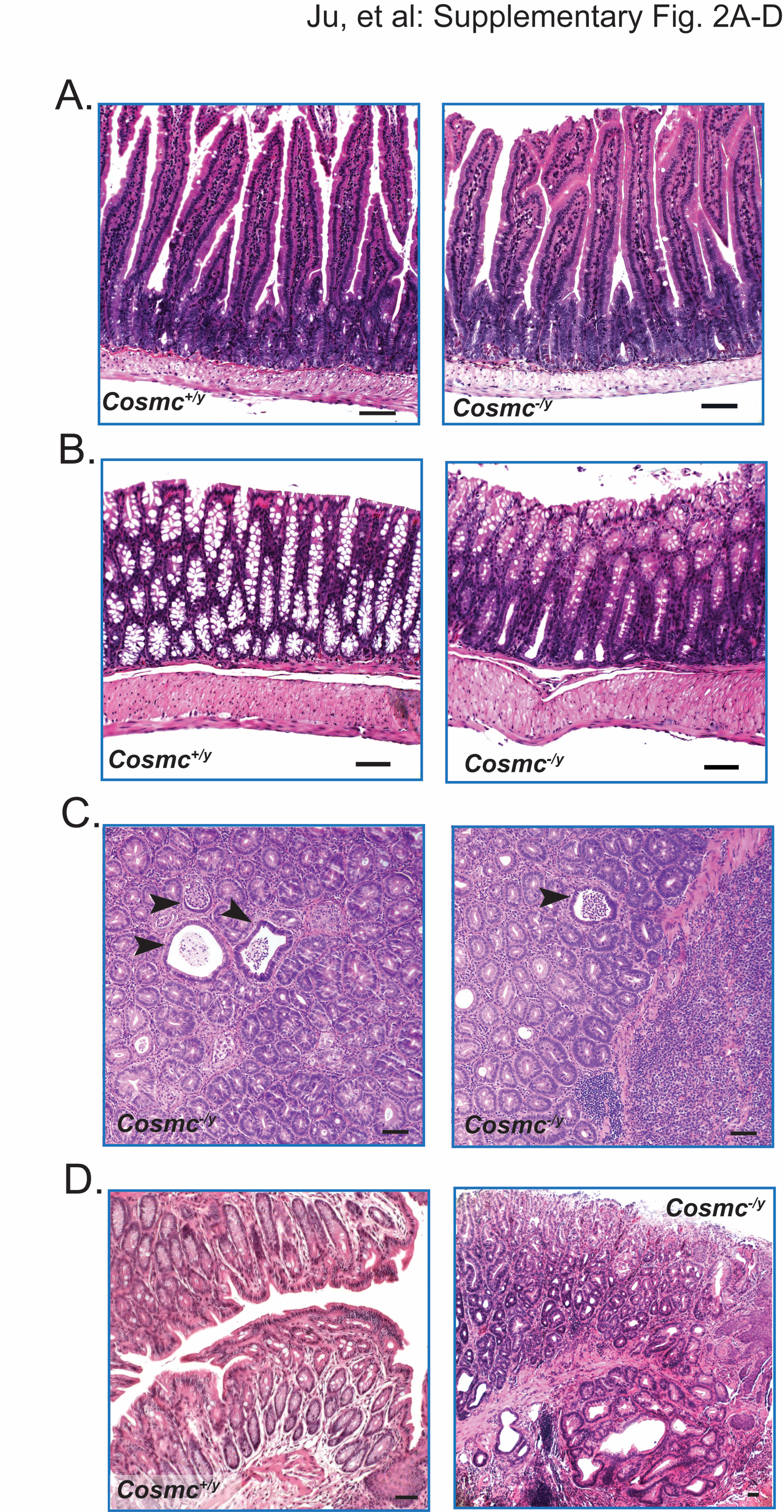


**Supplemental Figure 3**


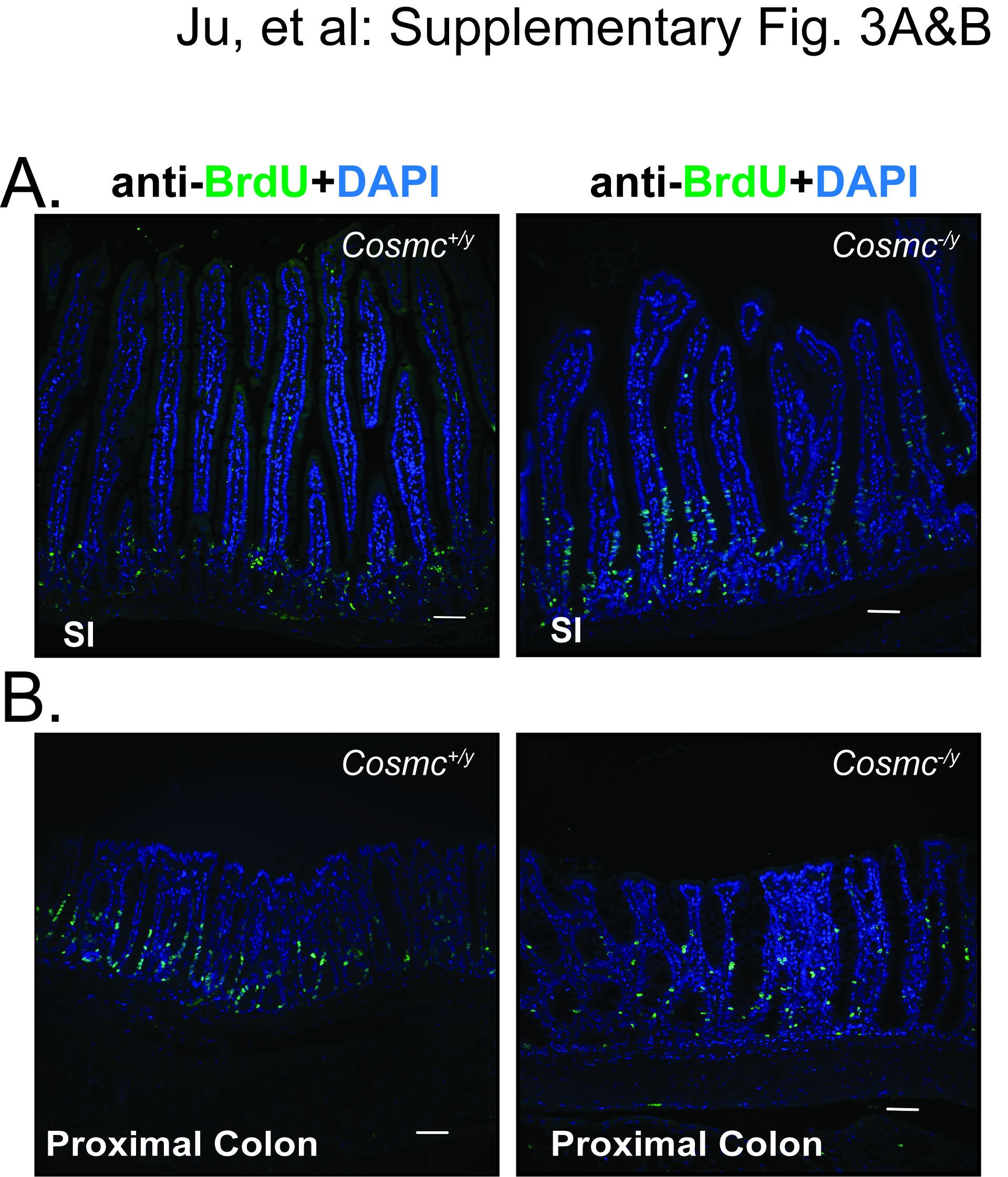


**Supplemental Figure 4**


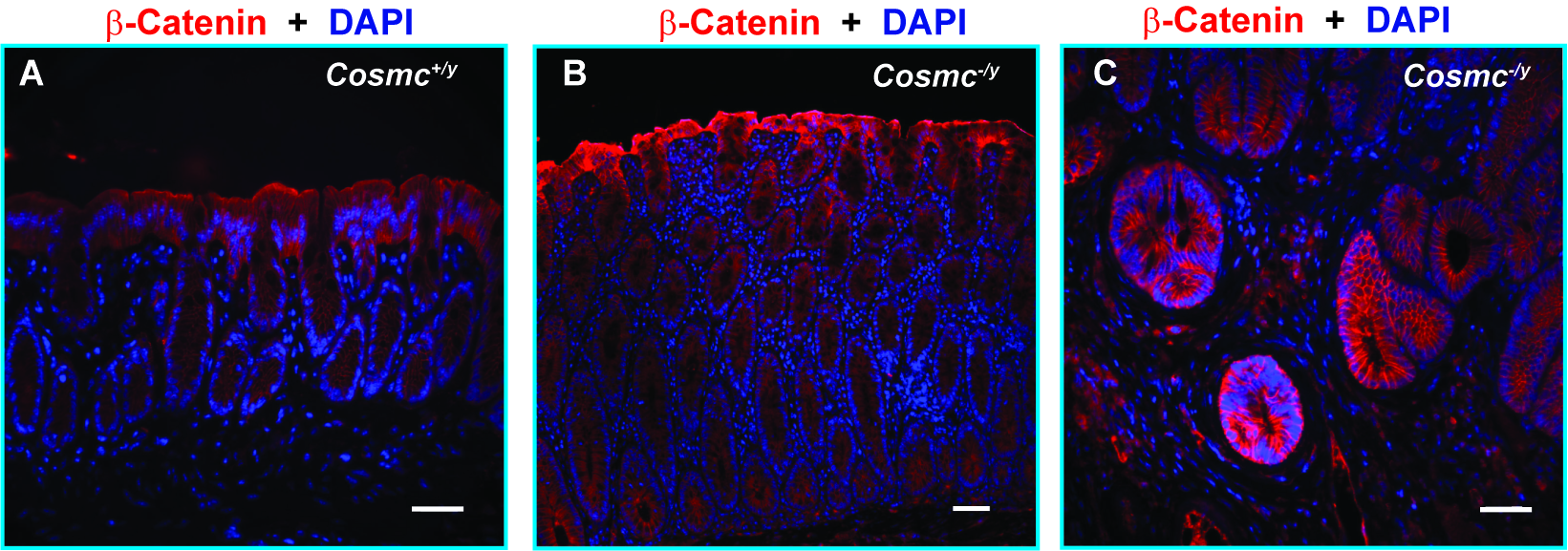

Supplement: Figures S1-S4 and Tables S1-S3 [file mmc1.docx]
